# USE OF REHABILITATION SERVICES AMONG OLDER CANCER SURVIVORS IN THE NATIONAL HEALTH AND AGING TRENDS STUDY

**DOI:** 10.1093/geroni/igae098.3921

**Published:** 2024-12-31

**Authors:** Min-Hui Huang, Thomas Chung

**Affiliations:** University of Michigan-Flint, Flint, Michigan, United States; University of Michigan-Flint, Flint, Michigan, United States

## Abstract

Older cancer survivors (OC) have higher risks of physical impairments than non-cancer older adults (NC). Cancer rehabilitation utilization remains a challenge. This study examined rehabilitation usage in OC using the National Health and Aging Trends Study (NHATS) round 12 (2022) dataset. Objectives were: 1) estimate rehabilitation prevalence in OC and NC; 2) identify factors associated with rehabilitation in each group; 3) compare rehabilitation duration, areas, and outcome between OC and NC. Community-dwelling adults ≥65 years with rehabilitation and cancer diagnosis responses were included (OC: N=653, age=78.7±7.33 years; NC: N=5168, age=79.9±7.63 years). General linear models (Objectives 1 and 2), Chi-square tests and t-tests (Objective 3) were conducted in IBM-SPSS version 28. Rehabilitation prevalence in the past year was similar between groups (OC: 26%; NC: 23%). Pain (OC: p=0.037; NC: p=0.004), higher education (OC: p< 0.001; NC: p< 0.001), and lower physical function (OC: p< 0.001; NC: p< 0.01) were linked to rehabilitation use in both groups. Unique factors linked to rehabilitation included low energy (p=0.030) for OC, and impaired lower-body strength (p=0.037) and balance (p=0.050) for NC. OC had longer rehabilitation durations (2.2±0.94 vs. 2.1±0.80 months, p< 0.001) and were more likely to rehabilitate for low energy (p=0.004), falls (p=0.040), walking inside (p=0.035) and outside the home (p=0.027). More OC (81.1%) reported meeting rehabilitation goals than NC (70.1%)(p=0.010). Despite similar rehabilitation prevalence between groups, distinct factors linked to rehabilitation usage in OC highlight the need for tailored programs. Positive rehabilitation outcomes in OC support more rehabilitation referrals to improve physical function.

